# Food characterization in the omics era: current advances, analytical approaches, and future perspectives

**DOI:** 10.3389/fnut.2026.1899127

**Published:** 2026-08-17

**Authors:** Muhammad Suhail Ibrahim, Wajahat Azeem, Hafiz Arbab Sakandar, Darragh Curran, Ali Asad Yousaf, Ioannis Zabetakis

**Affiliations:** 1 Institute of Food and Nutritional Sciences, PMAS-Arid Agriculture UniversityRawalpindi, Pakistan; 2 Department of Plant Pathology, PMAS-Arid Agriculture UniversityRawalpindi, Pakistan; 3 Department of Microbiology and Immunology, National University of Medical Sciences, Rawalpindi, Pakistan; 4 Department of Chemical Sciences, University of Limerick, Limerick, Ireland; 5 Bernal Institute, University of Limerick, Limerick, Ireland; 6 Health Research Institute (HRI), University of Limerick, Limerick, Ireland

**Keywords:** artificial intelligence, food authentication, food safety, foodomics, genomics, lipidomics, metabolomics, multi-omics

## Abstract

Food characterization is fundamental to ensuring food quality, safety, authenticity, traceability, and nutritional value in increasingly complex global food systems. Conventional analytical approaches often provide limited information on the molecular complexity of food matrices, necessitating the adoption of advanced, high-throughput technologies. Omics sciences, including genomics, transcriptomics, proteomics, metabolomics, lipidomics, and microbiomics, have emerged as powerful tools for comprehensive food characterization by enabling the large-scale analysis of genes, transcripts, proteins, metabolites, lipids, and microbial communities. The integration of these disciplines within the framework of foodomics has transformed food research from conventional assessments to systems-level investigations of food composition, functionality, and biological effects. This review provides a comprehensive overview of the major omics platforms and their applications in food quality evaluation, authenticity verification, safety monitoring, traceability, and nutritional assessment. Particular emphasis is placed on the role of multi-omics approaches in identifying molecular biomarkers associated with food origin, processing, adulteration, and health-promoting attributes. Advances in analytical technologies, including high-resolution mass spectrometry, nuclear magnetic resonance spectroscopy, and next-generation sequencing, are discussed in the context of foodomics workflows. Furthermore, the integration of chemometrics, machine learning, and artificial intelligence with omics datasets is examined as a means of improving data interpretation, predictive modeling, and real-time decision-making throughout the food supply chain. The review also highlights current challenges related to data integration, standardization, analytical reproducibility, and bioinformatics infrastructure, while outlining emerging opportunities in precision nutrition, digital food systems, and sustainable food production. By synthesizing recent developments across multiple omics disciplines, this review underscores the pivotal role of foodomics and multi-omics strategies in advancing comprehensive food characterization and supporting the development of safer, more authentic, and nutritionally optimized food systems.

## Graphical abstract

**Figure:**
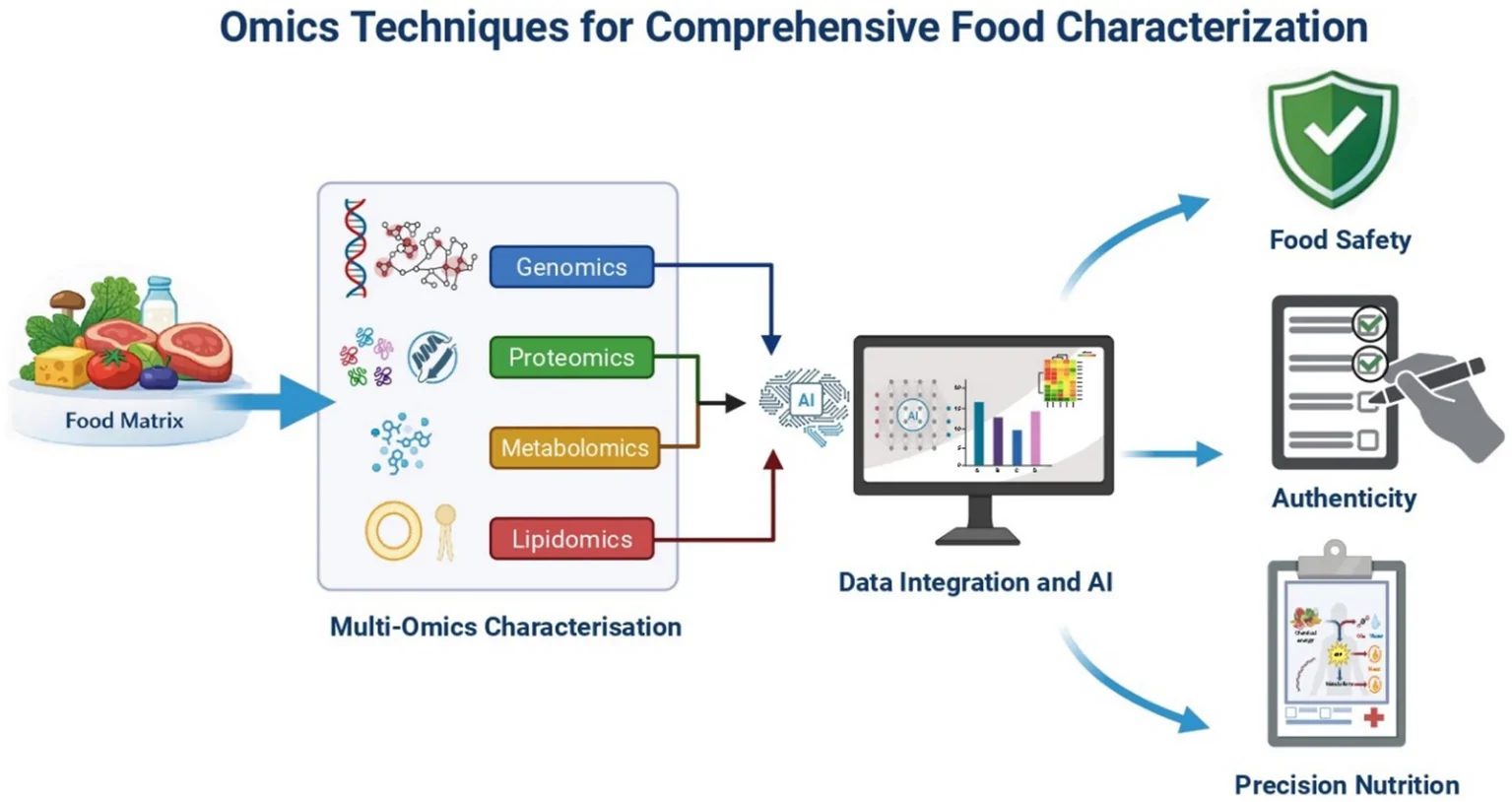

## 1 Introduction

The comprehensive characterization of food is essential for ensuring its quality, safety, authenticity, and nutritional value. Modern food systems are increasingly complex, driven by globalization, industrialization, and growing demands for transparency across production and supply chains (1–3). Consumers are no longer focused solely on sensory attributes such as taste, color, and texture; they also seek detailed information on nutritional composition, safety, sustainability, and provenance (4, 5). To meet these challenges, food scientists and technologists are increasingly relying on omics techniques, which are high-throughput molecular approaches originally developed for biomedical research, to provide deeper insights into the structure, function, and composition of food at a systems level (6–8).

Omics technologies encompass a range of powerful analytical platforms, including genomics, transcriptomics, proteomics, metabolomics, lipidomics, and foodomics, which collectively enable a holistic understanding of complex food matrices (9–11). By integrating these techniques with bioinformatics and chemometrics, researchers can decode complex molecular interactions within foods, track biomarkers of authenticity, detect contaminants, and explore the impact of bioactive compounds on human health (12, 13). These methods are fundamentally changing how food is studied, moving beyond conventional proximate analysis and standard chemical assays towards data-intensive, systems-level approaches.

**Figure 1:**
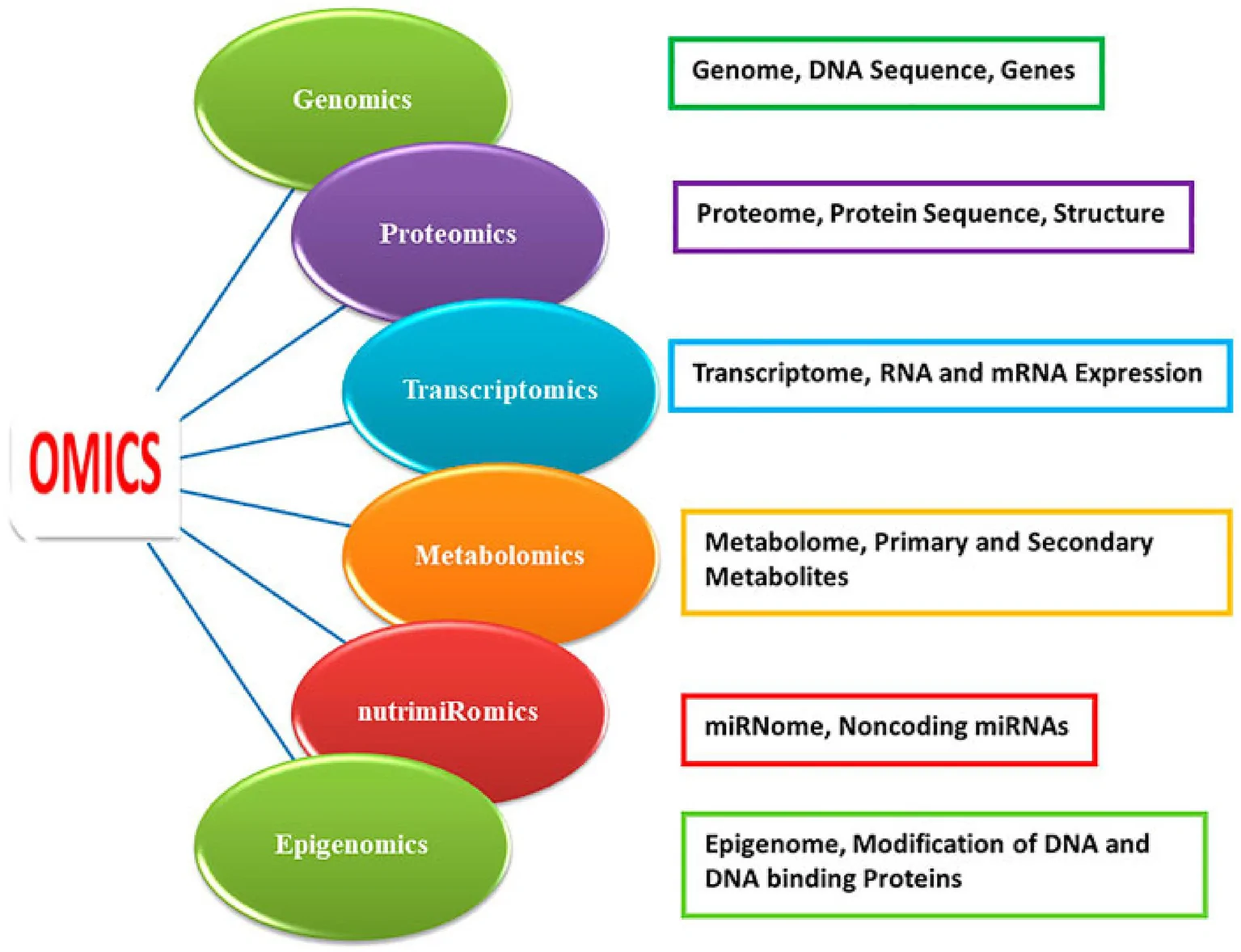
Application of omics techniques in various biological systems.

A central driver for the application of omics in food characterization is the increasing demand for food safety and authenticity. High-value commodities including olive oil, honey, dairy products, and wine are being adulterated, mislabeled, and counterfeited as part of global food fraud, which is believed to cost the industry billions per year. Omics-based tools have proven invaluable for detecting subtle molecular fingerprints that differentiate authentic products from adulterated ones. For instance, proteomics can identify specific protein biomarkers unique to a species or processing method, while metabolomics enables the generation of metabolic profiles that reveal geographical origin or adulteration (14). Genomic tools such as DNA barcoding and next-generation sequencing are widely employed to trace food origin and species identification, particularly in meat and seafood authentication (15). The relevance of omics in nutritional and functional food research is equally significant. By recording differences in amino acids, fatty acids, vitamins, and bioactive substances, metabolomics and lipidomics offer comprehensive insights into the nutritional composition of food. These molecular signatures help to understand how foods contribute to health and wellbeing, particularly in the context of functional foods and nutraceuticals. Omics-based approaches also support the development of precision nutrition strategies, where individualized dietary recommendations are informed by metabolic responses, genetic background, and gut microbiome interactions (16–18). Such developments align with global health initiatives aimed at preventing diet-related chronic diseases through tailored nutrition strategies. Although several reviews have examined individual omics disciplines, including genomics, proteomics, metabolomics, and lipidomics, a comprehensive synthesis of their collective application to food characterization remains limited. Moreover, the rapid emergence of multi-omics integration, artificial intelligence, machine learning, and advanced bioinformatics has significantly expanded the capabilities of food characterization beyond traditional analytical frameworks. It is important to distinguish between the analytical platforms that enable molecular detection and the omics approaches themselves. Techniques such as mass spectrometry, chromatography, NMR spectroscopy, and other spectroscopic methods serve as instrumental foundations—they can operate in targeted or untargeted modes but do not constitute omics *per se*. Omics approaches are defined by their systems-level, high-throughput investigation of entire molecular classes (genome, transcriptome, proteome, metabolome, lipidome). This review focuses on omics methodologies and their integrative application, while acknowledging the analytical platforms that facilitate them (Figure 1).

## 2 Literature search strategy

This narrative review was conducted by systematically searching the PubMed, Web of Science, Scopus, and Google Scholar databases for peer-reviewed articles published between 2015 and 2026. The search strategy employed combinations of the following keywords: “foodomics,” “food omics,” “food genomics,” “food proteomics,” “food metabolomics,” “food lipidomics,” “multi-omics food,” “omics food authentication,” “omics food safety,” “machine learning food omics,” and “chemometrics food analysis.” Additional articles were identified through cross-referencing of bibliographies from relevant reviews and seminal papers. Priority was given to recent advances (2020–2026) while including foundational studies where necessary for conceptual clarity. Studies were selected based on relevance to omics-based food characterization, technical innovation, and applicability to food quality, safety, authenticity, or nutrition. Only articles published in English were considered.

## 3 From traditional food characterization to systems-level omics

Food characterization has long relied on established physical, chemical, microbiological, and sensory methods that remain foundational to quality control and regulatory compliance. Physical characterization, encompassing texture analysis, colorimetry, rheology, and microscopy, quantifies structural and mechanical properties that directly influence consumer acceptance and processing behavior. Chemical characterization, through proximate analysis, chromatography (GC, HPLC, ion chromatography), and spectroscopic techniques (UV–Vis, FTIR, NMR, MS), provides detailed compositional data on nutrients, contaminants, and bioactive compounds. Microbiological and DNA-based methods ensure safety through pathogen detection and species authentication, while sensory evaluation captures aspects of human perception that instrumental methods cannot replicate. These conventional approaches, reviewed extensively elsewhere, have served the food industry reliably for decades. However, they are inherently reductionist: each method targets predefined analytes or properties, often in isolation, and cannot capture the emergent complexity of food matrices as dynamic biological systems. Each traditional technique is briefly described below.

### 3.1 Physical characterization

**Figure 2:**
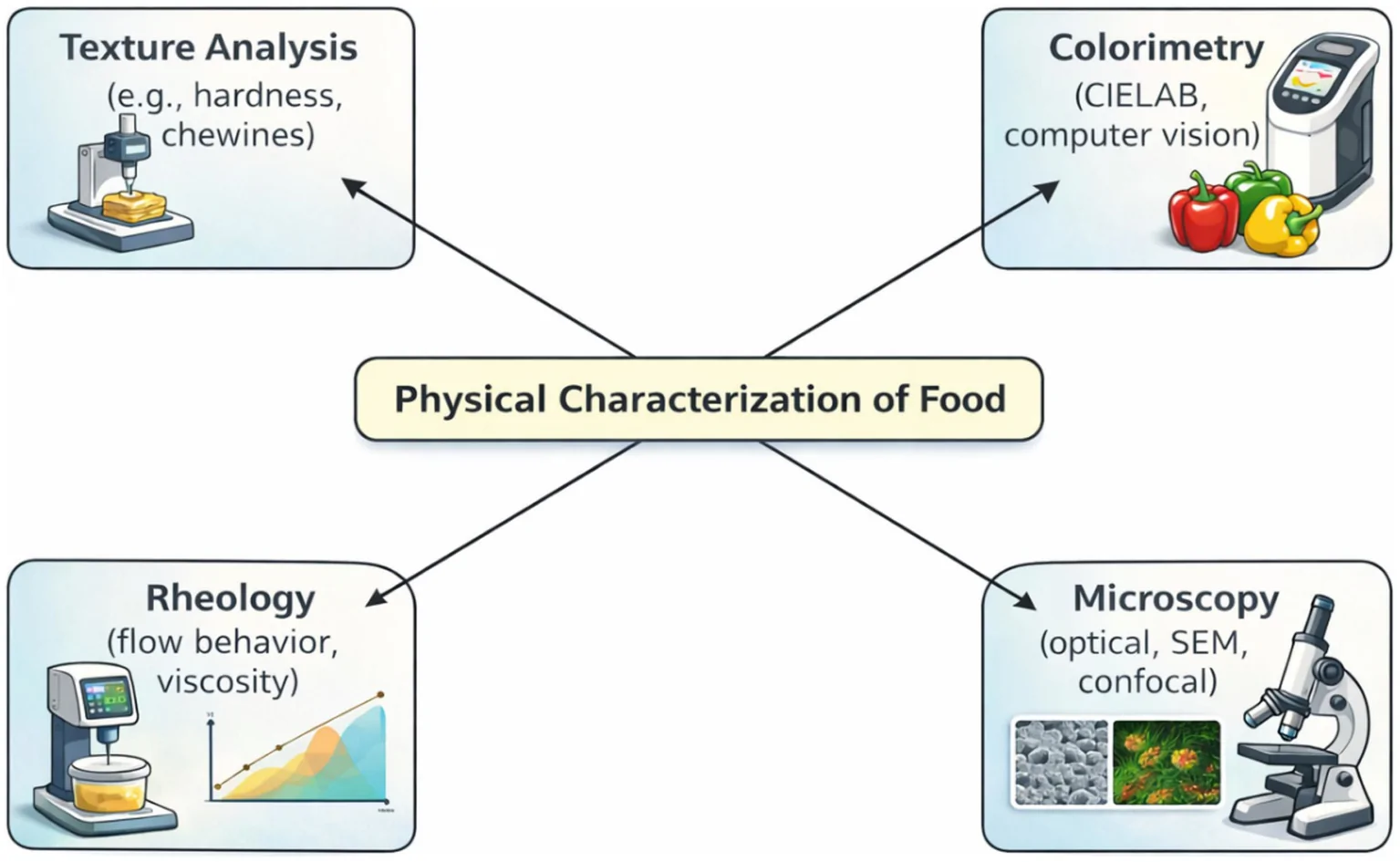
Schematic of physical characterization methods in food.

These techniques provide critical insights into the structural and mechanical properties of food, complementing molecular-level analyses obtained through omics approaches. Among the physical characteristics of any food item, texture is a key quality attribute influencing consumer acceptance. Texture analysis involves quantifying mechanical properties such as hardness, cohesiveness, springiness, and chewiness using specialized instruments like texture analyzers (19, 20). Techniques such as the Texture Profile Analysis (TPA) simulate the action of the jaw during mastication, providing a comprehensive profile of textural attributes (21–23). Texture evaluation is particularly important in bakery products, dairy products, fruits, and meat, where structural integrity and mouthfeel directly affect marketability (24) (Figure 2).

Color is one of the most important sensory attributes of food, influencing both consumer perception and purchase decisions. Colorimetry, the science of quantifying and describing color, plays a crucial role in food characterization by providing objective measurements of food appearance (25–27). Unlike subjective sensory evaluations, instrumental colorimetry ensures reproducibility and precision, making it indispensable in quality control, product development, and authenticity assessment (28).

The most widely applied system in food science is the CIE L*a*b* color space, where *L* indicates lightness, *a* represents the red-green axis, and *b* represents the yellow-blue axis (25, 27, 29). These values can be converted into additional metrics, such as chroma and hue angle, which offer deeper insights into food quality. Modern advancements extend beyond handheld colorimeters to computer vision systems that capture spatial distribution of color in foods. These systems integrate imaging techniques with color analysis, providing detailed assessments of surface uniformity and defects (30). Furthermore, hyperspectral imaging combines spectral and spatial data, enabling simultaneous evaluation of color, composition, and authenticity (31–34). Another important physical property is rheology, the science of deformation and flow of matter, which plays a vital role in food characterization as it directly influences processing behavior, product stability, and consumer perception (35, 36). Rheological measurements provide insights into texture, mouthfeel, spreadability, and pourability, which are key determinants of consumer acceptance. For example, the creaminess of yogurt, the chewiness of dough, and the spreadability of margarine are strongly linked to their rheological profiles (37, 38). Most foods are non-Newtonian, showing shear-thinning, thixotropic, or viscoelastic properties (39). Rheological parameters such as viscosity, yield stress, storage and loss moduli (G′ and G″) are determined using instruments like rotational rheometers and texture analyzers. These measurements are essential in optimizing processing conditions, predicting shelf life, and designing novel formulations (36). Similarly, coupling rheology with imaging techniques such as confocal microscopy has provided deeper insights into the relationship between food microstructure and mechanical behavior (40). These integrative approaches are particularly important in designing functional foods and plant-based alternatives, where texture must mimic animal-derived products (41–43).

Furthermore, rheology is increasingly being used in 3D food printing, where extrusion-based technologies rely on precise control of flow and deformation behavior to create customized food structures (35, 44–47).

Additionally, microscopy is a fundamental technique in food characterization, enabling the visualization and analysis of structural, compositional, and functional properties at micro- and nanoscales. Since many food properties, such as texture, flavor release, and stability, are determined by their microstructure, microscopy provides critical insights that complement physicochemical and rheological measurements (48–50). Several microscopy techniques such as light microscopy (LM), confocal laser scanning microscopy (CLSM), transmission electron microscopy (TEM), scanning electron microscopy (SEM), Cryo-SEM, Cryo-TEM and atomic force microscopy (AFM) are widely used in food characterization (51–58). Moreover, combining microscopy with spectroscopy, such as Raman or FTIR, has enabled correlative imaging that simultaneously provides structural and chemical information (59, 60).

### 3.2 Chemical characterization

#### 3.2.1 Proximate analysis

Proximate analysis determines moisture, ash, protein (Kjeldahl or Dumas), fat (Soxhlet), and carbohydrates (by difference), providing foundational compositional data (61) (Table 1).

**Table 1:** Major chemical characterization techniques in food science.

Technique	Target analyte	Application	Advantage	Limitation	References
GC–MS	Volatiles, fatty acids	Flavor profiling, lipid oxidation	High sensitivity	Requires derivatization	(134, 135)
HPLC	Phenolics, vitamins	Nutritional analysis	Widely applicable	Sample prep intensive	(136, 137)
FTIR	Bulk composition	Rapid fingerprinting	Minimal sample prep	Lower specificity	(90, 91)
NMR	Metabolites	Structural analysis	Non-destructive	High cost	(138, 139)

#### 3.2.2 Chromatographic techniques

##### 3.2.2.1 Gas chromatography

GC separates volatile and semi-volatile compounds with high resolution and sensitivity. GC–MS enables precise identification of aroma compounds, while GC-IRMS verifies geographical origin (62–66). GC × GC improves separation of complex mixtures, and portable GC systems support on-site monitoring (67–70).

##### 3.2.2.2 High-performance liquid chromatography

HPLC analyzes non-volatile and thermally labile compounds, e.g., vitamins, phenolics, mycotoxins, and proteins. Unlike GC’s volatility constraint (71–75), HPLC-MS/MS detects trace contaminants (e.g., aflatoxins and ochratoxin A) with high selectivity (76, 77). UHPLC offers faster analysis with reduced solvent use, and HRMS coupling enables untargeted metabolomics (74, 78–81).

##### 3.2.2.3 Ion chromatography

IC quantifies ionic nutrients (e.g., Na^+^, K^+^, Ca^2+^, Cl^−^, NO₃^−^) and additives (e.g., nitrites and nitrates) in a single run (82). IC-MS enhances trace contaminant detection (e.g., perchlorates and bromates), while microbore systems improve sustainability (83).

#### 3.2.3 Spectroscopic techniques

##### 3.2.3.1 UV–vis spectroscopy

UV–Vis spectroscopy is rapid and cost-effective for quantifying chromophoric compounds including phenolics, anthocyanins, and carotenoids, as well as antioxidant capacity (DPPH, FRAP, ABTS) (84–86). Handheld devices and HPLC-UV coupling enable on-site and selective detection (87).

##### 3.2.3.2 Infrared spectroscopy (IR)

FTIR provides molecular fingerprints for adulteration detection (e.g., foreign oils in olive oil and milk fraud) and monitors oxidation, denaturation, and carbohydrate transformations. NIR quantifies major constituents (moisture, fat, protein, fiber) for online process control. Hyperspectral imaging adds spatial resolution for defect detection (88–91).

##### 3.2.3.3 Nuclear magnetic resonance

NMR elucidates molecular structure and quantifies metabolites without extensive sample preparation. ^1^H/^13^C NMR profiles link wine and coffee to their origin and sensory quality; qNMR enables absolute quantification without calibration curves (92–95). TD-NMR rapidly measures moisture, fat, and protein in meat and dairy. Portable benchtop NMR supports on-site authenticity testing.

##### 3.2.3.4 Mass spectrometry

MS offers unmatched sensitivity and selectivity for trace analysis. LC–MS/MS screens pesticide residues; HRMS enables untargeted metabolomics. IRMS and FT-MS verify geographical origin and detect adulteration in olive oil, honey, and wine (1, 96–98). Ambient ionization (DESI-MS, DART-MS) allows direct food analysis without preparation, and portable devices enable rapid field testing (Table 2).

**Table 2:** Comparison of chemical characterization techniques in food.

Technique	Main applications	Advantages	Limitations	Recent cases	References
GC/GC–MS	Volatiles, fatty acids, pesticides, aroma	High resolution, sensitive, reproducible	Requires derivatization for non-volatiles	Aroma profiling, pesticide détection	(134, 152)
HPLC	Polyphenols, vitamins, amino acids, toxins	Wide applicability, accurate quantitation	Expensive solvents, longer analysis time	Polyphenol and vitamin analysis	(137)
IC	Organic acids, minerals, ionic compounds	Sensitive for ionic species	Limited to ionic analytes	Mineral content in beverages	(140)
UV–Vis	Pigments, antioxidants, food colorants	Rapid, cost-effective, simple	Poor specificity, overlapping signals	Antioxidant activity assays	(141, 142)
FTIR/NIR	Authenticity, adulteration, compositional analysis	Fast, non-destructive, minimal prep	Requires calibration, chemometric models	Olive oil adulteration detection	(91, 94, 95)
NMR	Metabolomics, untargeted profiling	Structural elucidation, minimal prep	Expensive, less sensitive than MS	Wine metabolomics	(139)
MS	Trace compounds, toxins, proteomics	Ultra-sensitive, highly specific	High cost, technical complexity	Mycotoxin and proteome profiling	(143)

### 3.3 Biological and molecular characterization

DNA-based techniques provide high specificity for species identification, fraud detection, and pathogen screening, as genetic material remains stable even after processing (99). PCR, qPCR, and multiplex PCR detect animal and plant DNA in processed foods for meat verification and allergen identification (100). DNA barcoding (mitochondrial COI, chloroplast *rbcL*) authenticates seafood and herbal products. NGS enables simultaneous detection of multiple species in complex matrices, uncovering hidden adulterants in olive oil, honey, milk, and meat (101). For food safety, PCR and qPCR identify *Salmonella*, *Listeria monocytogenes*, and *E. coli* O157: H7 within hours (102, 103), while digital PCR and LAMP further enhance sensitivity for low-concentration pathogens. GMO detection relies on standardized DNA assays for regulatory compliance (101). Emerging CRISPR-based biosensors and portable sequencers are advancing real-time, on-site applications (104). Microbiological testing ensures food safety and quality by detecting pathogens, spoilage organisms, and beneficial microbes (105). Additionally, culture-based methods (plate counts, selective agar, biochemical assays) remain the gold standard for confirming *Salmonella*, *Listeria monocytogenes*, *E. coli* O157: H7, and *Staphylococcus aureus* (106), but require 24–72 h and may miss viable but non-culturable (VBNC) organisms. Molecular methods, including PCR, qPCR, and LAMP, reduce detection times to hours and provide higher sensitivity. NGS and metagenomics enable comprehensive microbial community profiling without cultivation, identifying pathogens, spoilage organisms, and probiotics (107–109). Similarly, rapid methods such as immunoassays, flow cytometry, and biosensors provide near real-time results. Nanotechnology and CRISPR-Cas-based biosensors support on-site pathogen detection, enhancing HACCP implementation in food processing systems. Enumeration of lactic acid bacteria and *Bifidobacterium* ensures proper labeling of fermented products such as yogurt, kefir, and kombucha. Moreover, digital integration with AI and IoT-enabled sensors enables proactive risk management, predictive food safety, and continuous monitoring, thereby reducing the reliance on end-product testing.

### 3.4 Data-driven characterization

Data-driven characterization integrates statistical analysis, machine learning, and computational modeling to extract patterns from large datasets, enabling faster predictions than conventional approaches. In food science, this approach addresses the complexity of food matrices through advanced data-processing pipelines (Table 3).

**Table 3:** Data driven characterization.

Technique	Application	Example	References
PCA (principal component analysis)	Data reduction, classification	Differentiating wine origins	(144, 145)
SVM (support vector machine)	Authentication	Detecting adulterated olive oil	(146, 147)
Neural networks	Pattern recognition	Predicting sensory scores	(148, 149)
Random forest	Contaminant detection	Identifying pesticide residues	(150)

### 3.5 Chemometrics

Chemometrics applies mathematical and statistical methods to chemical data, enabling pattern recognition and predictive modeling in food authentication, quality control, and safety monitoring (110). Principal Component Analysis (PCA), Partial Least Squares-Discriminant Analysis (PLS-DA), and Hierarchical Cluster Analysis (HCA) classify products by origin, variety, or production method and have been used to authenticate olive oil, honey, wine, and coffee (111). For spectroscopic data (FTIR, NIR, Raman, NMR), chemometric tools reduce noise, extract features, and build calibration models for quantitative analysis of moisture, protein, fat, and sugars (110). In food safety, PLS regression and artificial neural networks (ANN) are used to quantify pesticide residues, mycotoxins, and heavy metals (112). Hybrid models combining chemometrics with AI enable robust real-time monitoring in digital food systems.

### 3.6 Machine learning and artificial intelligence

Machine learning (ML) and artificial intelligence (AI) automate the interpretation of multidimensional datasets generated by spectroscopy, chromatography, proteomics, and genomics (113). Supervised models—including support vector machines (SVM), random forest (RF), and ANN—classify foods by origin or detect adulteration using spectral fingerprints (110, 153). Convolutional neural networks (CNNs) support image-based analysis for defect detection and ripeness grading (113). For food safety, ML integrates chromatographic and biosensor data to predict pesticide, mycotoxin, and microbial contamination, thereby enhancing early warning systems (114). In metabolomics, AI identifies biomarkers distinguishing fresh from processed foods, while sensory prediction models link volatile compounds and rheological data to flavor, aroma, and texture. The synergy between AI and chemometrics, together with the integration of IoT and blockchain integration, is driving smart food characterization platforms for real-time decision-making and traceability (113, 155, 156).

## 4 Major omics disciplines in food characterization

Omics technologies have emerged as powerful tools for comprehensive food characterization, providing detailed molecular insights into food composition, quality, authenticity, safety, and nutritional value (115, 116). By enabling the simultaneous analysis of thousands of biomolecules, including genes, transcripts, proteins, metabolites, lipids, and microbial communities, omics approaches have significantly enhanced our understanding of food systems and their interactions with human health. The integration of genomics, transcriptomics, proteomics, metabolomics, lipidomics, and microbiomics has transformed conventional food analysis from targeted assessments into holistic and systems-based investigations.

The following sections delineate the core omics disciplines, their analytical foundations, and their transformative applications in modern food science, building upon and transcending the capabilities of traditional characterization. Table 4 provides a comparative overview.

**Table 4:** Role of omic disciplines in food characterization.

Omics	What is measured/focus	Key technologies	Food-relevant insights	References
Genomics	The full DNA content: species genome, gene variants, microbial community DNA (metagenomics)	Next-generation sequencing (short-read, long-read), PCR, DNA microarrays	Species identification, cultivar/variety discrimination, detection of GMO, microbial profiling in fermented foods, adulteration, geographical origin determination	(9)
Transcriptomics	RNA transcripts (mRNAs, non-coding RNAs)—what genes are being expressed under given conditions	RNA-seq, microarrays, qPCR	Response of plants (or microbes) to environmental stresses; how gene expression changes with processing, storage; understanding allergen expression; yield, flavor/aroma biosynthesis	(10)
Proteomics and peptidomics	All proteins/peptides in a sample: structure, post-translational modifications, abundance	Mass spectrometry (shotgun proteomics, targeted proteomics), 2D-PAGE, gel electrophoresis, LC–MS/MS, quantification techniques (iTRAQ, SILAC etc.)	Identify allergens, enzymes affecting shelf life, proteins responsible for texture or flavor, authentication (e.g., species in meat, dairy), degradative changes during storage	(11, 12)
Metabolomics	Small molecule metabolites (primary and secondary)—sugars, organic acids, polyphenols, amino acids etc.	NMR spectroscopy, GC–MS, LC–MS, CE-MS, often targeted or untargeted metabolomics	Flavor profile, nutritional value, detection of contaminants, metabolic fingerprinting for origin/authenticity, tracking metabolic changes during ripening, processing or storage	(13, 14)
Lipidomics	The lipid species: fatty acids, glycerolipids, phospholipids, sterols, sphingolipids etc.	LC–MS, GC–MS, ion mobility MS, direct infusion mass spectrometry, MS imaging, NMR	Understanding fat composition, oxidation, rancidity; nutritional quality (e.g., omega-3 vs. saturated fats), origin/authenticity (e.g., types of oils), flavor compounds generated via lipid degradation	(15)
Epigenomics and other emerging omics	Modifications that do not change DNA sequence but affect expression (DNA methylation, histone modifications), microbiome transcriptomics/metaproteomics/metametabolomics, exposomics etc.	Bisulfite-seq, ChIP-seq, ATAC-seq, metatranscriptomics, etc.	How environment or storage influences gene regulation, phenotypic changes in plants induced by growing conditions, microbes’ regulation in fermented foods, adaptive responses to stresses etc.	(16–18)

### 4.1 Genomics and transcriptomics

Genomics examines the complete DNA content of organisms, enabling species identification, cultivar discrimination, detection of genetically modified organisms (GMOs), and microbial community profiling through metagenomics. Next-generation sequencing (NGS) technologies, including short-read and long-read platforms, have transformed food authentication by providing high-resolution genetic fingerprints (9). DNA barcoding targeting mitochondrial or chloroplast genes remains essential for seafood and herbal product verification. Transcriptomics captures the dynamic expression of RNA transcripts under specific conditions, revealing how genes respond to environmental stresses, processing parameters, and storage conditions (10). In food science, transcriptomics elucidates allergen expression patterns, flavor biosynthesis pathways, and stress-induced metabolic shifts in plants and fermentation microbes.

### 4.2 Proteomics and peptidomics

Proteomics characterizes the entire protein complement of a food sample, including post-translational modifications that alter functionality and allergenicity. Mass spectrometry-based approaches, such as shotgun proteomics, targeted selected reaction monitoring (SRM/MRM), and data-independent acquisition (DIA), enable high-throughput identification and quantification of proteins in complex matrices (11, 12). Two-dimensional gel electrophoresis (2-DE) and liquid chromatography–tandem mass spectrometry (LC–MS/MS) remain foundational techniques. In food characterization, proteomics identifies species-specific protein markers for authentication (e.g., detecting non-bovine milk in cheese), tracks enzymatic changes during ripening and storage, and profiles allergenic proteins in milk, eggs, peanuts, and seafood (117, 118). Proteomics, the large-scale study of proteins and their functions, has emerged as a vital approach in food science for characterizing nutritional content, detecting allergens, ensuring authenticity, and improving food safety. Since proteins are directly linked to biological function and can undergo modifications during processing, proteomics provides insights that complement DNA- and metabolite-based analyses (117). The use of advanced technologies such as mass spectrometry (MS), two-dimensional gel electrophoresis (2-DE), and liquid chromatography–tandem MS (LC–MS/MS) has enabled high-throughput identification and quantification of proteins in complex food matrices. A major application of proteomics in food is allergen detection and characterization. Food allergies are a growing public health concern, and proteomic methods allow precise identification of allergenic proteins in products such as milk, eggs, peanuts, soy, and seafood. High-resolution MS enables not only the detection of allergenic proteins but also the characterization of post-translational modifications that may affect allergenicity (118). Proteomics-based allergen profiling is increasingly being incorporated into regulatory frameworks and risk assessment protocols. Proteomics also plays a crucial role in food authenticity and fraud detection. By establishing protein fingerprints, researchers can distinguish between species, verify geographical origin, and identify adulteration in products such as meat, fish, dairy, and plant-based foods. For example, proteomic profiling can differentiate between bovine and non-bovine milk in cheese production or detect undeclared meat species in processed foods. In nutritional and functional food research, proteomics provides detailed information about bioactive peptides, enzymes, and protein modifications that influence digestibility and health benefits. This is especially important in dairy and cereal products, where processing conditions such as heat treatment or fermentation significantly alter protein structure and functionality. Another significant application is food safety and pathogen detection. Proteomic methods are employed to identify protein markers of microbial contamination, toxins, and spoilage, allowing earlier intervention in the food chain. Shotgun proteomics and targeted approaches have been used to detect bacterial and fungal contaminants in dairy and meat products with high sensitivity (119). Recent advances in label-free quantification, targeted proteomics (SRM/MRM), and data-independent acquisition (DIA) are improving reproducibility and accuracy in food proteomics. Furthermore, integration with bioinformatics and multi-omics approaches (proteomics, genomics, and metabolomics) is enhancing our understanding of food quality, traceability, and health impacts. In summary, proteomics is a versatile and powerful tool in food science, offering applications in allergen detection, authenticity verification, nutritional research, and safety monitoring. As technologies evolve, proteomics will continue to expand its role in ensuring food quality and consumer protection.

### 4.3 Metabolomics

Metabolomics profiles small-molecule metabolites—including sugars, organic acids, amino acids, polyphenols, and volatiles—providing a functional readout of biological activity. Unlike genomics or proteomics, metabolomics reflects the net result of gene expression and protein activity, making it highly sensitive to environmental and processing variables (13, 14). Untargeted metabolomics using nuclear magnetic resonance (NMR) spectroscopy, gas chromatography–mass spectrometry (GC–MS), and liquid chromatography-mass spectrometry (LC–MS) generates comprehensive metabolic fingerprints for origin authentication and quality assessment. Targeted metabolomics quantifies specific compounds such as mycotoxins, vitamins, or bioactive polyphenols. Metabolomics has proven particularly powerful for tracing geographical origin, detecting adulteration in wine, honey, and olive oil, and monitoring metabolic changes during ripening and processing.

### 4.4 Lipidomics

Lipidomics focuses on the comprehensive analysis of lipid species, including fatty acids, glycerolipids, phospholipids, sterols, and sphingolipids. As a distinct yet complementary discipline to metabolomics, lipidomics addresses the structural diversity and biological significance of lipids in food matrices (15). LC–MS, GC–MS, and ion mobility mass spectrometry enable detailed lipid profiling, while direct-infusion MS and MS imaging provide information on the spatial distribution of lipids. Food lipidomics informs nutritional quality (e.g., omega-3-to-saturated fatty acid ratios), tracks lipid oxidation and rancidity, verifies oil authenticity through characteristic lipid signatures, and identifies flavor compounds generated via lipid degradation pathways.

### 4.5 Foodomics

The convergence of multiple omics disciplines has given rise to the concept of foodomics, first introduced in 2009, which represents a comprehensive framework for studying food and nutrition at the molecular level. Foodomics integrates information obtained from genomics, transcriptomics, proteomics, metabolomics, and other omics platforms to investigate the complex relationships between food components, biological systems, and human health (120, 121). Within this framework, lipidomics has gained increasing attention due to its ability to characterize bioactive lipids and elucidate their roles in inflammation, metabolic regulation, and chronic disease prevention. Foodomics studies have demonstrated how bioactive compounds present in fruits, vegetables, cereals, and other plant-based foods can modulate gene expression and metabolic pathways associated with oxidative stress and inflammation. Consequently, foodomics is playing a crucial role in the discovery of novel biomarkers, the development of functional foods, and the advancement of personalized nutrition strategies.

Foodomics plays a significant role in nutritional and functional food research. Metabolomics and transcriptomics are used to investigate how bioactive compounds in foods, such as polyphenols, carotenoids, and peptides, interact with human metabolic pathways (122). These approaches help uncover the molecular mechanisms by which functional foods contribute to health benefits, including antioxidant activity, anti-inflammatory effects, and gut microbiota modulation (123). Such insights support the development of precision nutrition strategies tailored to individual metabolic responses.

In food safety, foodomics enables the detection of contaminants, toxins, and pathogens at a molecular level. Advanced proteomic and metabolomic tools can identify specific biomarkers of microbial spoilage or chemical hazards, providing an early warning system for potential risks in the food chain. Integration of foodomics with machine learning further enhances predictive capabilities, enabling real-time monitoring and risk assessment in complex supply chains.

The future of foodomics lies in its integration with systems biology and big data analytics. Multi-omics approaches, supported by bioinformatics, allow researchers to model the interactions between food components and human metabolic networks. This contributes to improved food quality and authenticity, as well as personalized dietary recommendations and sustainable food production. Foodomics is revolutionizing food science by enabling comprehensive, molecular-level insights into food quality, authenticity, safety, and health impacts. Its interdisciplinary nature makes it a cornerstone for the next generation of food research and innovation. This integrative approach combines genomics, proteomics, metabolomics, and transcriptomics for holistic food profiling (124–126).

## 5 Emerging omics: microbiomics, epigenomics, and exposomics

Microbiomics characterizes microbial communities in food and production environments through metagenomic, metatranscriptomic, and metaproteomic approaches. It supports fermentation optimization, spoilage prediction, and pathogen detection (127, 128, 154). Epigenomics examines heritable modifications, i.e., DNA methylation and histone modifications, that regulate gene expression without altering the DNA sequence, offering insights into how environmental conditions and diet influence phenotypic outcomes (16–18). Exposomics, an emerging frontier, seeks to characterize the totality of environmental exposures affecting food systems and human health. These disciplines are increasingly being integrated into multi-omics frameworks to capture the full complexity of food-biological interactions.

## 6 Integration challenges

The complexity of food matrices—diverse chemical compositions influenced by agricultural practices, environmental conditions, and processing—demands robust sample preparation, high-resolution instrumentation, and advanced bioinformatics pipelines. Multi-omics integration presents particular challenges, as harmonizing vast datasets across genomic, proteomic, metabolomic, and lipidomic layers requires standardized protocols, interoperable databases, and interdisciplinary collaboration among food scientists, chemists, bioinformaticians, and data scientists (129, 130, 151).

## 7 Applications in food systems

One of the most important applications of omics technologies is in food safety monitoring. The presence of chemical contaminants, pesticide residues, toxins, adulterants, and microbial pathogens remains a major challenge for the global food industry. Traditional analytical methods often suffer from limitations in sensitivity, specificity, and throughput, particularly when dealing with complex food matrices. In contrast, omics-based approaches, coupled with advanced analytical platforms such as mass spectrometry (MS), chromatography, and spectroscopic techniques, facilitate the high-throughput detection and identification of contaminants at trace levels. Proteomic and metabolomic profiling can reveal specific molecular signatures associated with microbial spoilage, toxin production, or contamination events, enabling earlier detection and improved risk assessment throughout the food supply chain (127, 131). Furthermore, microbiomics has provided unprecedented insights into food-associated microbial communities, contributing not only to pathogen surveillance but also to the optimization of fermentation processes and the development of novel food products (127, 128). In food quality assessment, omics approaches facilitate the identification of biomarkers associated with freshness, flavor, texture, shelf life, and processing conditions. These molecular markers enable manufacturers to maintain product consistency, optimize processing parameters, and improve customer satisfaction. In terms of food safety, foodomics techniques are widely used to detect contaminants, toxins, allergens, pathogenic microorganisms, pesticide residues, and antibiotic resistance markers with high sensitivity and accuracy, thereby reducing health risks and supporting regulatory compliance. Omics technologies also play a crucial role in food authenticity and traceability. They enable verification of geographical origin, species identification, genetic composition, and product integrity, helping to detect adulteration in high-value food products such as meat, dairy, olive oil, honey, and seafood. Consequently, these approaches contribute significantly to combating food fraud and protecting consumer confidence. In the field of nutrition, nutrigenomics and metabolomics provide valuable insights into the interactions between diet, genetics, and metabolism (Table 5).

**Table 5:** Specific applications where omics and foodomics are used.

Application area	Omics techniques often used	Examples of insights/outcomes
Authenticity/adulteration	Genomics, metabolomics, proteomics, lipidomics	Detecting substitution of expensive plants/herbs, meat species content, adulteration of oils (olive oil mixed with cheaper oils), geographic origin via metabolic fingerprints
Food quality and flavor	Metabolomics, lipidomics, proteomics, transcriptomics	Understanding flavor compound evolution during ripening or processing; lipid oxidation leading to off-flavors; enzymatic changes affecting texture, aroma enzymes expression regulation
Nutritional profiling and bioactive compounds	Metabolomics, lipidomics, proteomics	Identifying vitamins, antioxidants, secondary metabolites with health properties; profiling fatty acid composition; finding novel bioactives or nutraceuticals; understanding how processing impacts nutritional content
Food safety	Genomics (especially metagenomics), proteomics, metabolomics	Detecting pathogens / microbial contaminants; profiling toxin production; allergens detection; monitoring chemical contaminants and residues; evaluating safety after processing/storage
Effect of processing and storage	Multi-omics (metabolomics + proteomics + lipidomics)	How heat, pH, drying, freezing, packaging affects compositional changes; degradation products; stability of vitamins, lipids; what reactions happen during storage (oxidation, Maillard reactions)
Traceability and geographical origin	Metabolomics, genomics (often via plant/animal-variety genetics), profiling signatures	Using metabolite / isotope / genetic markers to certify origin, avoid fraud, label protection (PDO, PGI etc.)
Allergenicity and health risks	Proteomics, transcriptomics, lipidomics, epigenomics	Identifying allergenic proteins; understanding expression regulation of allergen genes; lipid dysregulation in response to allergens; monitoring epigenetic modifications possibly induced by dietary components

These approaches support the development of personalized nutrition strategies and functional foods tailored to individual health requirements. Foodomics further contributes to the discovery and characterization of bioactive compounds, antioxidants, and other health-promoting ingredients that may help prevent chronic diseases and improve overall wellbeing. Recent advances in computational sciences have further expanded the capabilities of omics technologies in food characterization. The integration of AI and ML with omics datasets has enabled the analysis of highly complex and multidimensional data generated by modern analytical platforms. AI-driven algorithms can identify hidden patterns, classify food products with high accuracy, and predict quality attributes, authenticity markers, and safety risks. For example, deep learning models applied to proteomic and metabolomic datasets have demonstrated remarkable performance in detecting food adulteration, assessing product quality, and predicting shelf-life characteristics (4, 132, 133). Such developments are paving the way for real-time monitoring systems and smart food production networks, thereby supporting the digital transformation of food systems.

## 8 Advantages and limitations of omics approaches

Despite these significant advances, several challenges continue to limit the widespread implementation of omics technologies in food characterization. Food matrices are inherently complex and heterogeneous, with their composition being influenced by genetic factors, environmental conditions, agricultural practices, postharvest handling, and processing techniques. Such complexity necessitates robust sample preparation protocols, highly sensitive analytical instrumentation, and sophisticated bioinformatics tools for data processing and interpretation. Moreover, the integration of datasets generated from different omics platforms remains a major challenge due to differences in data structure, scale, and analytical methodologies. Therefore, effective multi-omics data integration requires advanced computational approaches and close collaboration among food scientists, analytical chemists, bioinformaticians, statisticians, and data scientists (129, 130) (Table 6).

**Table 6:** Advantages and limitations of omics approaches.

Advantage	Limitation/challenge
High throughput; can detect many molecules at once (many metabolites, many proteins)	Food matrices are messy; presence of interfering compounds; extraction biases; need for expensive instrumentation
Ability to detect unexpected or unknown compounds (untargeted)	Identification of unknowns is difficult; libraries are incomplete; data interpretation is complex
Sensitivity (particularly MS)	Dynamic range issues: very abundant vs. very low abundance compounds; sometimes low-abundance compounds get lost
Ability to track biological responses (e.g., expression, regulation) across omics layers (gene→protein→metabolite)	Omics data integration is nontrivial; consistency issues; regulatory levels do not always match (mRNA ≠ protein ≠ metabolite)
Helps in authenticity, safety, nutritional value, flavor etc.	Cost, time, need of specialized personnel; reproducibility; standardization; bioinformatics infrastructure needed

## 9 Future trends and perspectives

Looking ahead, food characterization is expected to increasingly rely on multi-omics integration and systems biology approaches. The combination of genomic, transcriptomic, proteomic, metabolomic, lipidomic, and microbiomic data with AI-driven analytical frameworks will enable the development of comprehensive models that describe food quality, safety, functionality, and health impacts. Such integrated approaches have the potential to improve food traceability, strengthen supply chain transparency, support sustainable food production systems, and facilitate consumer access to reliable product information. Furthermore, continuous advancements in high-resolution mass spectrometry, nuclear magnetic resonance (NMR) spectroscopy, next-generation sequencing (NGS), and computational analytics are expected to enhance the accuracy, speed, and applicability of omics-based food characterization. Overall, omics technologies have revolutionized food characterization by providing unprecedented molecular-level insights into food composition, authenticity, safety, and nutritional functionality. The integration of advanced analytical techniques, bioinformatics, artificial intelligence, and systems biology has established foodomics as a transformative paradigm in modern food science. As analytical capabilities continue to evolve, omics-driven approaches are expected to play an increasingly central role in addressing contemporary challenges related to food quality, food security, personalized nutrition, and sustainable food systems.

## 10 Conclusion

Omics technologies have transformed food characterization by providing comprehensive molecular-level insights into food composition, authenticity, safety, and nutritional quality. Through the integration of genomics, transcriptomics, proteomics, metabolomics, lipidomics, and foodomics, these approaches enable precise detection of biomarkers, contaminants, and bioactive compounds, while enhancing food traceability and quality assessment. The incorporation of artificial intelligence and advanced data analytics further strengthens their predictive and monitoring capabilities. Although challenges related to data integration, standardization, and implementation remain, ongoing advances in analytical platforms and bioinformatics are expanding their accessibility and application. As multi-omics approaches continue to evolve, they are expected to play a pivotal role in supporting food innovation, personalized nutrition, sustainable production, and consumer confidence in modern food systems.
